# Model-Assisted Control of Flow Front in Resin Transfer Molding Based on Real-Time Estimation of Permeability/Porosity Ratio

**DOI:** 10.3390/polym8090337

**Published:** 2016-09-08

**Authors:** Bai-Jian Wei, Yao-Chen Chuang, Kai-Hong Wang, Yuan Yao

**Affiliations:** Department of Chemical Engineering, National Tsing Hua University, Hsinchu 30013, Taiwan; padget790201@gmail.com (B.-J.W.); ycchuang@mx.nthu.edu.tw (Y.-C.C.); expipiisminus1@gmail.com (K.-H.W.)

**Keywords:** polymer composite, resin transfer molding, flow control, permeability estimation, meta-model, cascade control

## Abstract

Resin transfer molding (RTM) is a popular manufacturing technique that produces fiber reinforced polymer (FRP) composites. In this paper, a model-assisted flow front control system is developed based on real-time estimation of permeability/porosity ratio using the information acquired by a visualization system. In the proposed control system, a radial basis function (RBF) network meta-model is utilized to predict the position of the future flow front by inputting the injection pressure, the current position of flow front, and the estimated ratio. By conducting optimization based on the meta-model, the value of injection pressure to be implemented at each step is obtained. Moreover, a cascade control structure is established to further improve the control performance. Experiments show that the developed system successfully enhances the performance of flow front control in RTM. Especially, the cascade structure makes the control system robust to model mismatch.

## 1. Introduction

In resin transfer molding (RTM), the degree of resin impregnation into the fibrous fiber preform directly influences the final quality of fiber reinforced polymer (FRP) products. For achieving quality improvement, researchers have devoted their research efforts to active flow control in RTM, so as to direct the resin flow in a desired manner and ensure the saturation of the fibers. The commonly adopted strategy is to alter the shape of the flow front either by adjusting injection pressure (or resin flow rate) at each injection gate [1,2,3,4,5,6,7,8,9,10,11] or by localized heating [12,13,14,15,16]. As pointed out in [17], the usage of localized heating is limited to resins with a long gelatinization time at elevated temperatures. Therefore, this paper mainly focuses on the former type of methods.

The feasibility of the flow control by adjusting injection pressure or resin flow rate has been demonstrated in literature. Sozer et al. [1] and Bickerton et al. [2] proposed an online strategic controller to influence the flow front pattern during mold filling and attempt to drive the process towards successful completion. In their method, numerical simulations are conducted offline to simulate the effects of each possible process disturbance, where the pressure distribution and the flow front locations are calculated as a function of time. In online implementation, the current process status is identified by embedded sensors, based on which appropriate adjustments of the flow rate of each gate are prescribed using a pre-constructed decision tree. It should be noted that the decision tree highly depends on expert experience. Later, in a two paper series [3,4], Hsiao and Advani as well as Devillard et al. integrated a flow sensing network and a flow actuation network to achieve satisfactory flow control. According to the genetic algorithm (GA) optimization results based on a pre-defined disturbance database, a binary on-off control is conducted at each gate and vent to adjust the resin flow. The completeness of the database is a critical factor for the success of such controller. To avoid the dependence on expert experience or large amount of historical data which may not be available in real industrial applications, Nielsen and Pitchumani [5] developed a model-based control framework, which acquires the flow fronts to use a charge-coupled device (CCD) camera and uses an artificial neural network (ANN) as a real-time process simulator for flow control. ANN is a kind of data-driven model, whose structure is inspired by biological neural networks. It is usually used to estimate or approximate functions that are generally unknown. In [5], the ANN was built based on the simulated data provided by a numerical process model, which relates the current flow front and the flow rate at each injection gate to the flow front at the next control step. By using this model, the future flow front can be predicted at each sampling time point. With the aid of the ANN model, the optimal combination of the flow rates at each step was calculated by conducting optimization. Later, Nielsen and Pitchumani [6] proposed another controller which searches for the optimal flow rates in a pre-determined schedule set based on the output of a numerical process model. Lawrence et al. [8] developed another model-based flow controller which uses flow simulations and GA to optimize the flow rate for each gate. A similar idea was adopted by Modi et al. [9]. In their paper, the injection gates are under on-off control. In online implementation, the optimal gate combination is selected based on numerical simulations.

Although the existing model-based flow control strategies have shown their effectiveness, some shortcomings still exist. First, the controller performance is easily affected by the model mismatch. The permeability of fiber preform is an especially important process parameter which varies with the preform architectures and directly determines the saturation of resin in fiber as well as the final product quality. Therefore, this parameter should be accurately specified in the simulation model used in flow control. However, most existing methods do not involve real-time permeability estimation. Instead, the preform permeability is usually assumed to be a known constant in the numerical simulators, which is not true. In practice, even the same kind of fiber made by the same manufacturer may have significant variations in permeability values. In addition, it is also observed that the fiber preform permeability values often vary for different mold shapes and fiber handling conditions. Nielsen and Pitchumani [7] made an attempt to solve this problem. In their control strategy, a fuzzy logic model is built based on a large number of input-output data generated by an RTM simulator, which provides estimates of the local pressure gradients. Then, local permeability is estimated by substituting the pressure gradients, the flow velocities, and the resin viscosity into the Darcy’s law. A potential risk of their method is that the mismatch between the real permeability and its estimate may still exist since the fuzzy logic model is built solely based on simulation data. Li et al. [18] developed an integrated RTM design and control system based on a three-step permeability estimation method named gas-assisted real-time assessment of permeability (GRASP). First, the mold is injected with the low flow rate gas. The corresponding pressure distribution is measured using a transducer array mounted in the mold. In the second step, the gas permeability is calculated by substituting the pressure profile into the equations of mass balance and Darcy’s law. Third, by referencing a gas-resin permeability correlation model, the permeability of the fiber preform when liquid resin is used is derived. As discussed in [19], GRASP is mainly suited to the applications with high fiber volume fractions. In addition, the control strategy proposed in [18] is open-loop, which is not robust to unpredicted disturbances.

In addition, most existing work aims to finding an optimal flow rate through each injection gate. However, the equipment for resin flow rate control is usually more expensive than those used for injection pressure control. In order to minimize costs for capital investment of equipment, an efficient flow control system by manipulating the injection pressure is desired. Due to the nonlinear and time-varying relationship between the pressure and the flow front velocity, the flow control in RTM is not an easy task. The conventional proportional–integral (PI) controller with fixed parameters or the on-off controller cannot achieve high precision control results. Mogavero et al. [20] proposed a nonlinear controller for achieving flow control by adjusting the injection pressure, where a process parameter related to the permeability is estimated in an adaptive way. As shown in their paper, the choice of the adaptation rate affects the control performance. Berg and Voller [21] developed another method under the assumption that the resin flows in a one-dimensional tube with unit area. Instead of searching for optimal setting of flow rates, Lee et al. [10] directly manipulated the injection pressure of each gate to let the actual flow front similar to the numerical simulation result. However, the detailed algorithm for automatic control is not provided in their paper. Restrepo et al. [11] also proposed a method to control the flow front with the injection pressure. However, the control algorithm does not take the variations in permeability into consideration.

In this paper, a model-assisted control strategy of flow front in resin transfer molding is developed. In the proposed method, the ratio between the equivalent permeability and the porosity (named permeability/porosity ratio in the following of this paper) of the fiber preform is calculated in real time, using the information acquired by a visualization system. The estimates are then substituted into a meta-model constructed with a radial basis function (RBF) network on the basis of process simulation data. In doing this, the model mismatch on permeability can be avoided. According to the optimization results based on the meta-model, the injection pressure can be adjusted online to control the velocity of the flow front. It should be noted that the control performance may also be affected by the mismatch in other process parameters besides permeability, e.g., fiber density, fluid viscosity, etc. To solve such problems, a cascade flow control structure is established to further improve the control system. The effectiveness of the proposed control strategy is illustrated using experiments. Although the experiments were conducted on an RTM system with one injection gate and one vent, the proposed method can be extended to control multi-gate systems. In fact, with satisfactory flow control on each injection gate, the shape of resin flow front can be adjusted to avoid defects in the final products.

## 2. Equipment and Instrumentation

**Figure 1:**
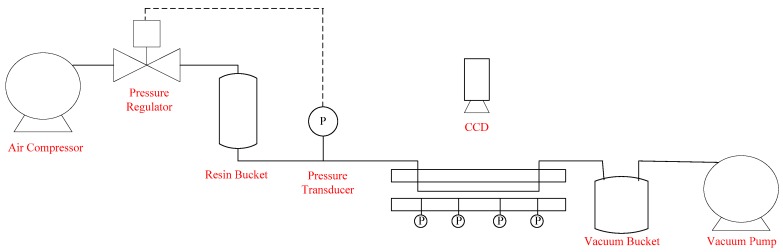
Piping and instrumentation diagram (P&ID) chart of experimental setup.

**Figure 2:**
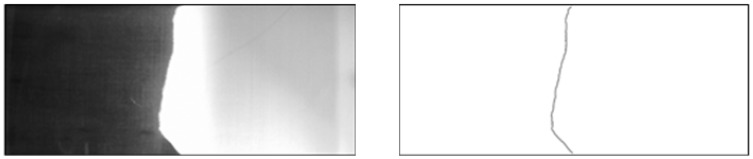
Instantaneous image of resin flow before and after image processing.

In this study, the flow front information is acquired by a visualization system comprised of a CCD camera (Allied Vision, Exton, PA, USA) and a mold with a transparent upper plate (Yong-Jia Mold, New Taipei, Taiwan). This upper plate is made of acrylic plastic, while the lower plate of the mold is made of aluminum. The size of the mold cavity is 30 cm × 12 cm × 0.3 cm. There is one injection gate and one outlet vent located at either end of the upper plate. During molding, the resin in a bucket connected to the gate is pressurized by the compressed air and injected into the mold. In the meantime, vacuum assisted infusion is provided by a vacuum bucket and a vacuum pump installed at the outlet of the mold. Figure 1 shows the piping and instrumentation diagram (P&ID) of the experimental setup. The instantaneous images of resin flow are photographed by the CCD camera connected to an image acquisition (IMAQ) frame grabber card (National Instrument, Austin, TX, USA) installed in an industrial computer. After conducting image processing, the flow front positions are captured as illustrated in Figure 2. Different types of image processing techniques can be utilized for flow front identification, e.g., binarization based on image contrast, edge detection, and so on. In this paper, binarization was implemented. In the experiments, the injection pressure is adjusted by a pressure regulator (SMC Corporation, Tokyo, Japan). Therefore, the control objective of this paper is to maintain a desired flow front by changing the set points of the pressure regulator in real time. Specifically, the controlled variable is the flow front velocity/position along the line connecting the inlet gate to the vent.

In the literature, a number of methods have been developed to acquire flow front information besides the visualization system used in this paper, such as the dielectric sensors [22], the capacitive electrode array [23], and so on. These sensors can also be integrated to the proposed control system as alternatives to the CCD camera.

In addition, a pressure transducer array is mounted on the lower plate of the mold to collect the information of the pressure distribution. It should be noted that such information is not necessary for the control of flow front. However, it is a basis for local permeability estimation, which is helpful to understand the process characteristics. For more details about the local permeability estimation and the pressure transducer array, please refer to [24].

## 3. Methodology

### 3.1. Process Simulator

The process simulator used in this paper is an RTM simulation module (Moldex3D, Chupei, Taiwan) [25], which describes the behavior of resin flow in porous fiber structures by applying the finite volume method to the governing equations, including the continuity equation

(1) ∇⋅ρu=0

the Darcy’s law

(2) $$u=-\frac{1}{\eta }K‧▽P$$

and the energy equation

(3) $$\frac{\partial }{\partial t}(\rho C_{p}T)+\nabla \cdot (\rho uC_{p}T)=k\nabla^{2}T+\eta {\dot{\gamma }}^{2}+\phi \frac{d\alpha }{dt}\Delta H$$

In the above equations, **u** is the Darcy velocity vector, *P* denotes the pressure, **K** is the tensor of permeability, *η* is the viscosity of the resin, *T* denotes the temperature, *t* is the time index, *ρ* is the resin density, *k* is the thermal conductivity, *C_p_* is the specific heat, $\dot{\gamma }$ represents the share rate, *ϕ* is the porosity of the fiber preform, *α* is the conversion ratio, and Δ*H* denotes the heat generated by the curing reaction. The resin viscosity *η* is predicted by the curing kinetics coupled with the Castro-Macosko model:

(4) $$\frac{d\alpha }{dt}+u\cdot \nabla \alpha =\left(k_{1}+k_{2}\alpha^{m_{1}}\right)‧{\left(1-\alpha \right)}^{m_{2}}$$

(5) $$\eta =\frac{\eta_{0}{\left(\frac{\alpha_{g}}{\alpha_{g}-\alpha }\right)}^{C_{1}+C_{2}\alpha }}{1+{\left(\frac{\eta_{0}\dot{\gamma }}{\tau^{*}}\right)}^{1-n}}$$

where *m*_1_ and *m*_2_ are the reaction orders, *k*_1_ and *k*_2_ are the material constants, η_0_ is the zero shear rate viscosity, τ^*^ is the parameter that describes the transition region between zero shear rates and the power law region of the viscosity curve, *n* is the power law index, α_g_ is the conversion ratio at the gel point, and *C*_1_ and *C*_2_ are the fitting constants.

By specifying the material properties and the operation conditions, the behavior of resin flow can be visualized by the simulator, while the flow front profile at each time point can be recorded.

### 3.2. Online Estimation of Permeability/Porosity Ratio

As it is known, the permeability of fiber preform is an important parameter affecting the flow behavior of the resin. Precise estimation of this parameter is crucial to simulation accuracy. In this paper, the process model is utilized as a component of the real-time control system. Therefore, online estimation of this parameter is desired.

In recent years, a number of methods have been developed for permeability estimation [7,19,26,27,28,29,30,31]. However, as discussed in [24], most of these methods were designed for offline estimation, while some others are limited by the completeness of the historical database. Most recently, Wei et al. [24] proposed to achieve online permeability estimation by utilizing a visualization system and a pressure sensor array mounted in the mold. This method is for local permeability estimation, while the equivalent permeability of the fiber preform at a larger scale is more useful in real-time control of flow front. Hence, this method is not suited to the task in hand. It is not possible to calculate the equivalent permeability by a simple arithmetic mean of the local values in series, due to the fact that the permeability is not an additive variable.

Inspired by the works of Lee et al. [29] and Wei et al. [24], an online estimation method for the equivalent permeability is adopted here. The Darcy’s law (Equation (2)) can be simplified as Equation (6) by introducing three assumptions [32]: flow coordinates follow the principle direction of fiber, i.e., the *x*-axis; resin almost flows in a one-dimensional direction; the *z*-axis scale is neglected.

(6) $$u=-\frac{K}{\eta }\frac{dP}{dx}$$

Here, *u* is the Darcy velocity, *K* is the equivalent permeability in the range between the injection gate and the flow front, and (*dP*/*dx*) denotes the equivalent pressure gradient that can be estimated as:

(7) $$\frac{dP}{dx}=\frac{\Delta P}{L}$$

where Δ*P* and *L* are the pressure difference and the length between the flow front and the injection gate, respectively. The pressure on flow front is zero due to the vacuum-assisted condition. Therefore, Equation (7) can be further simplified to:

(8) $$\frac{dP}{dx}=-\frac{P^{0}}{L}$$

where *P*^0^ is the absolute injection pressure. In the experiments, the flow front velocity captured by the CCD camera is not the Darcy velocity *u* but the seepage velocity *v*, i.e., the actual velocity of resin flowing through the channels of the preform. The relationship between these two types of velocities can be described as:

(9) $$u=v\phi =\frac{dL}{dt}\phi$$

Substitution of both Equations (8) and (9) into Equation (6) leads to Equation (10):

(10) $$\frac{dL}{dt}\phi =\frac{K}{\eta }\frac{P^{0}}{L}$$

Taking the moving flow front and the variations in the injection pressure into consideration, the above equation becomes:

(11) $$\frac{dL_{t}}{dt}\phi =\frac{K_{t}}{\eta }\frac{P_{t}^{0}}{L_{t}}$$

where *t* is the index of time. By integration, the equivalent permeability *K_t_* estimated at time point *t* can be obtained as:

(12) $$K_{t}=\frac{L_{t}^{2}\phi \eta }{2\sum_{i=0}^{t}P_{i}^{0}T},$$

If the injection pressure at each sampling time point $P_{t}^{0}$, the current flow front position *L_t_*, *T* is the interval between two sampling time points, the fiber porosity *ϕ* and the resin viscosity *η* are known. However, in real applications, it is not reasonable to assume *ϕ* to be uniform, because permeability strongly depends on porosity. Fortunately, as shown in Equations (10)–(12), the resin flow behavior is actually affected by this ratio instead of only the permeability. Therefore, instead of estimating *K_t_*, the ratio between the equivalent permeability and the equivalent porosity, i.e., *R_t_* = *K_t_*/*ϕ_t_*, can be obtained using Equation (12) at each sampling time point. The permeability/porosity ratio *R_t_* summarizes the local properties for the fiber preform between the injection gate and the current flow front and is useful in the following flow front control step.

It should be noted that, in many RTM processes, the resin flow may not be one-dimensional. However, satisfactory control performance can be achieved by using the permeability/porosity ratio *R_t_*, especially when the cascade control structure that will be introduced in Section 3.4 is adopted.

### 3.3. Meta-Modeling

Although the simulator provides accurate prediction of the filling behavior of the resin, it is computationally intensive. Hence, it is not suited to online implementation and cannot be directly integrated into the real-time control system. A solution to such a problem is to use a meta-model. Also known as surrogate model, meta-model is a further abstraction of the simulation model. The basic concept of meta-modeling is to develop a relatively simple empirical model based on the data generated by the simulator. Since the meta-model is data-driven, it is usually computationally efficient in applications. Therefore, it can be used in place of the original simulator for analysis and optimization purposes [33].

**Figure 3:**
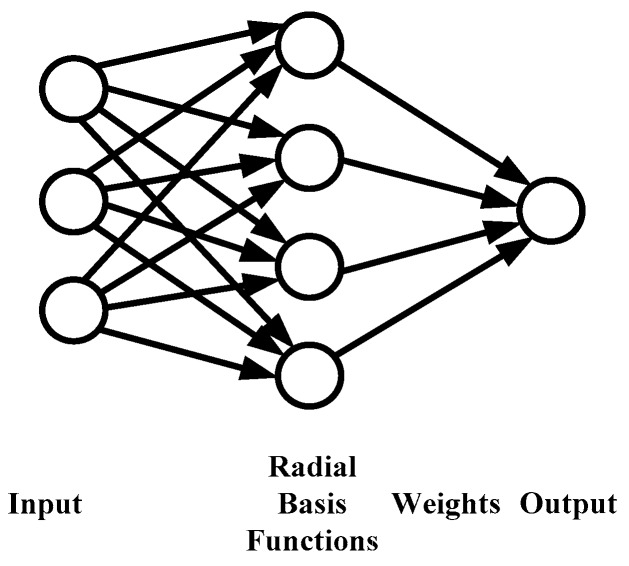
Illustration of RBF (radial basis function) network.

Here, due to its superiority in fast training and good interpolation approximation, the RBF network [34] is adopted to build the meta-model for the Moldex3D RTM module. As illustrated in Figure 3, there are typically three layers in an RBF network: an input layer, a hidden layer with a non-linear RBF activation function, and an output layer. The arrows in the figure symbolize the signal flow in the network. As a special type of ANN, the RBF network uses radial basis functions as activation functions, whose output is a linear combination of the radial basis functions of the inputs and neuron parameters. In an RBF network with a single output, the inputs are usually described as a vector **x**, while the output *ψ*(**x**) is a scalar function of the input vector:

(13) $$\psi (x)=\sum_{j=1}^{A}w_{j}\varphi_{j}(x)$$

where *A* is number of neurons in the hidden layer, $w_{j}$ is the output weight of the *j*-th neuron, and $\varphi_{j}$ denotes the activation function used by the *j*-th neuron. In other words, given an input vector **x**, the RBF network calculates the output *ψ*(**x**) using Equation (13). Different types of RBFs can be adopted to be the activation functions. A common choice is the Gaussian function:

(14) $$\varphi_{j}(x)=\exp(-\frac{||x-\mu_{j}|{|}^{2}}{2\sigma_{j}^{2}})$$

with the parameter σ*_j_* > 0, where **μ**_j_ is the center vector for the *j-*th neuron. The parameters *w_j_*, **μ**_j_, and σ*_j_* are determined by optimizing the fit between the model output and the measured data. With good training, the RBF network is capable of describing the complex nonlinear relationship between the inputs and the output.

To build a meta-model for the process simulator, the simulation data are collected under 275 different operating conditions by varying the injection pressure and the permeability/porosity ratio. In other words, 275 different batches are simulated for data generation purposes. In each batch, the injection pressure and the permeability are set to be constants. The observations recorded in these batches form the training dataset of the RBF network. It should be noted that with a proper design of computer experiments, it is possible to reduce the number of training data while achieving an RBF network model with similar precision. This issue is beyond the scope of this paper. More details of design of computer experiments can be found in the literature [35].

The inputs of the RBF network contain three variables, i.e., the current position of flow front *L_t_*, the current injection pressure $P_{t}^{0}$, and the permeability/porosity ratio *R_t_*_-1_ estimated at the previous sampling time point, while the flow front position *L_t_*_+1_ at the next time point is selected as the output. Thus, the resulting meta-model can be represented as:

(15) $$L_{t+1}=f\left(L_{t},P_{t}^{0},R_{t-1}\right)$$

Due to the interpolation ability of the RBF network, the meta-model can achieve satisfied predictions in a batch with varying injection pressure and equivalent permeability, although the training data are collected under several constant operating conditions.

### 3.4. Model-Assisted Control

The control objective is to vary the injection pressure $P_{t}^{0}$ so that the motion of the resin flow front follows a prescribed behavior. In particular, constant velocity control for flow front is considered in this paper for illustration. In this section, a model-assisted control strategy is developed.

**Figure 4:**
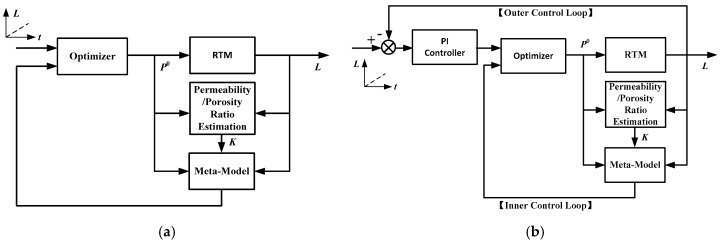
Diagrams of (**a**) the model predictive control system; (**b**) the cascade control system.

By integrating the RBF network meta-model, the real-time permeability/porosity ratio estimator, and an optimizer, a model predictive control (MPC) system is constructed, whose diagram is plotted in Figure 4a. In each step, the permeability/porosity ratio *R_t_*_−1_ is estimated using Equation (12), based on the flow front position *L_t_*_−1_ captured by the CCD camera and the injection pressure executed at the previous sampling time points. Then, the estimated *R_t_*_−1_ is substituted into the meta-model Equation (15) together with the current flow front position *L_t_*. By using an optimizer, the optimal value of the current injection pressure $P_{t}^{0}$ can be determined. The objective function of the optimizer is:

(16) $$\begin{array}{l} \underset{P_{t}^{0}}{\min}{\left[f\left(L_{t},P_{t}^{0},R_{t-1}\right)-\left(L_{t}+v^{\mathrm{sp}}T\right)\right]}^{2}+\lambda {\left[P_{t}^{0}-P_{t-1}^{0}\right]}^{2}\\ s.t.:\;P_{\min}\leq P_{t}^{0}\leq P_{\max} \end{array}$$

where *v*^sp^ is the set-point of the flow front velocity, *λ* is a weighting factor adjusting the trade-off between tracking performance and control effort, and *P*_min_ and *P*_max_ are the upper and lower bounds of the injection pressure, respectively.

In Equation (16), the meta-model plays an important role, whose accuracy is crucial to the control performance. When there is a model mismatch, the injection pressure calculated from Equation (16) may not be optimal, and offset may appear in the control results. There are several ways to eliminate the effects of model mismatch. For example, the RBF network model may be trained using the historical process data instead of the simulation data. In doing so, the mismatch between the simulator and the physical system does not affect the control performance. However, the RTM manufacturing process is often time-consuming and costly, hence it is usually difficult to collect enough historical data under different operating conditions for model training. Alternatively, the model migration technique [36] may be adopted to improve the accuracy of the meta-model using a small number of process data. Nevertheless, such method does not consider the unpredictable disturbances. When external disturbances during infusion exist, the filling behavior of resin will deviate from the model prediction. In this paper, a cascade control system structure as sketched in Figure 4b tilized to solve such problem, where an outer loop is added into the system to adjust the set-point of the MPC. Since the nonlinear and time-varying characteristics are largely compensated by the inner loop, a PI controller with fixed parameters is adopted in the outer control loop.

## 4. Results and Discussions

In this section, the effectiveness of the proposed control system is illustrated with the experiment results. First, the prediction accuracy of the process simulator and the meta-model is investigated with the comparison between the prediction results and the experimental data. Then, the feasibility and the limitations of the model-assisted control system without the cascade structure, i.e., the MPC, are shown through the experiments. At last, the cascade model-assisted control system is utilized to further improve the results of the MPC.

### 4.1. Process Simulator and Meta-Model

**Figure 5:**
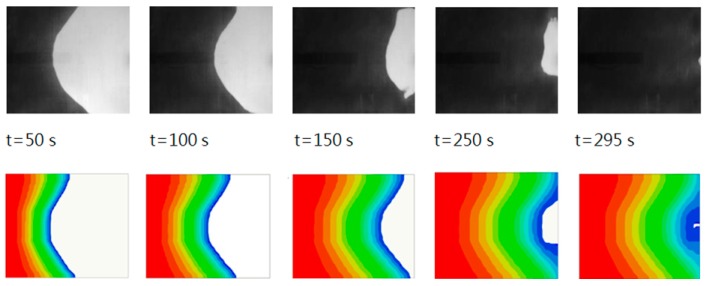
Comparison between the simulation results and the experimental data.

As introduced in Section 3.1, the RTM module developed by Moldex3D was utilized as the process simulator. Figure 5 shows the simulation results of a constant-pressure infusion experiment, where the upper plots display the real flow fronts captured by the CCD camera at the 50th, 100th, 150th, 250th, and 295th second respectively after the injection began, and the lower plots are the corresponding simulation results, where different colors indicate the flow front positions achieved at different sampling time points. The injection pressure was 1 bar. It is clear that with a proper parameter setting the simulation provided a good approximation of the experiment, confirming the reasonability of training the data-based RBF network model based on the process simulator.

**Figure 6:**
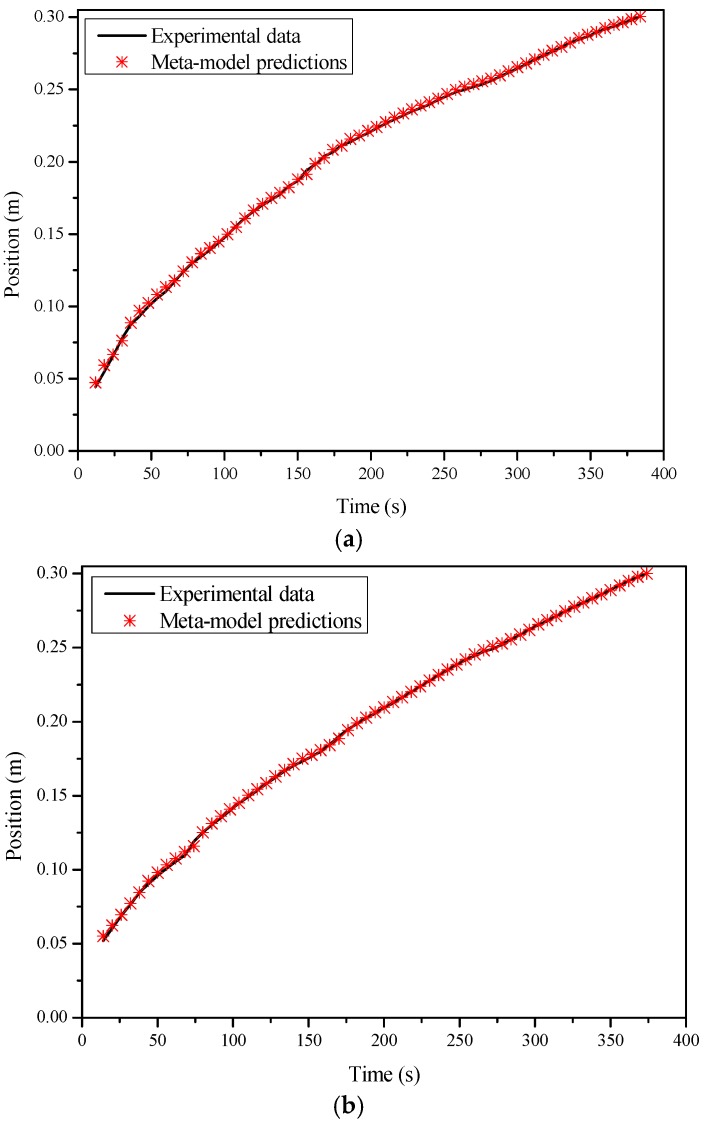
Comparison between the meta-model predictions and the results from (**a**) the first experiment; (**b**) the second experiment.

The accuracy of the meta-model is illustrated by comparing the prediction results with the outputs of the experimental data. In Figure 6, the outputs of the meta-model are compared with the data from the experiments. In the first experiment, the preform was made up of nine layers of glass fiber mats, whose porosity was about 54.6%. Constant-pressure infusion was conducted, where the injection pressure was set to be 1.2 bar. The permeability of the preform was also nearly constant, which was around 2.2 × 10^−10^ m^2^. The root mean squared error (RMSE) of this comparison is 5.8 × 10^−4^ m. Such a small value indicates that the meta-model effectively reflects the process characteristics, although it was built based on the simulation data. The second experiment was carried out under the condition of variable injection pressure, where the injection pressure was decreased by 0.02 bar per 18 s and changed from 1.3 to 0.88 bar. The preform was again made up of nine layers of fiber mats, whose permeability was about 2.5 × 10^−10^ m^2^. This time, the RMSE equals to 1.0 × 10^−3^ m, which is larger than previous values, but still very small.

### 4.2. Performance of the Model Predictive Controller

As discussed, the nonlinear and time-varying characteristics of the RTM process make the flow front control a difficult task that cannot be accomplished well by a binary on-off controller or a conventional PI controller with fixed parameters. For illustration, a PI controller was designed. To determine the controller parameters, a step test [37] was conducted when the flow front reached approximately half length of the mold. In this test, the injection pressure was increased from 0 to 2 bar in a short time, while the response of the velocity was recorded. Based on the collected data, a first-order with dead time model was built by using the method introduced in [37]. After *Z*-transform, the following discrete model was obtained:

(17) $$G(z)=\frac{1.6\times 10^{-3}}{z-0.56}z^{-1}$$

**Figure 7:**
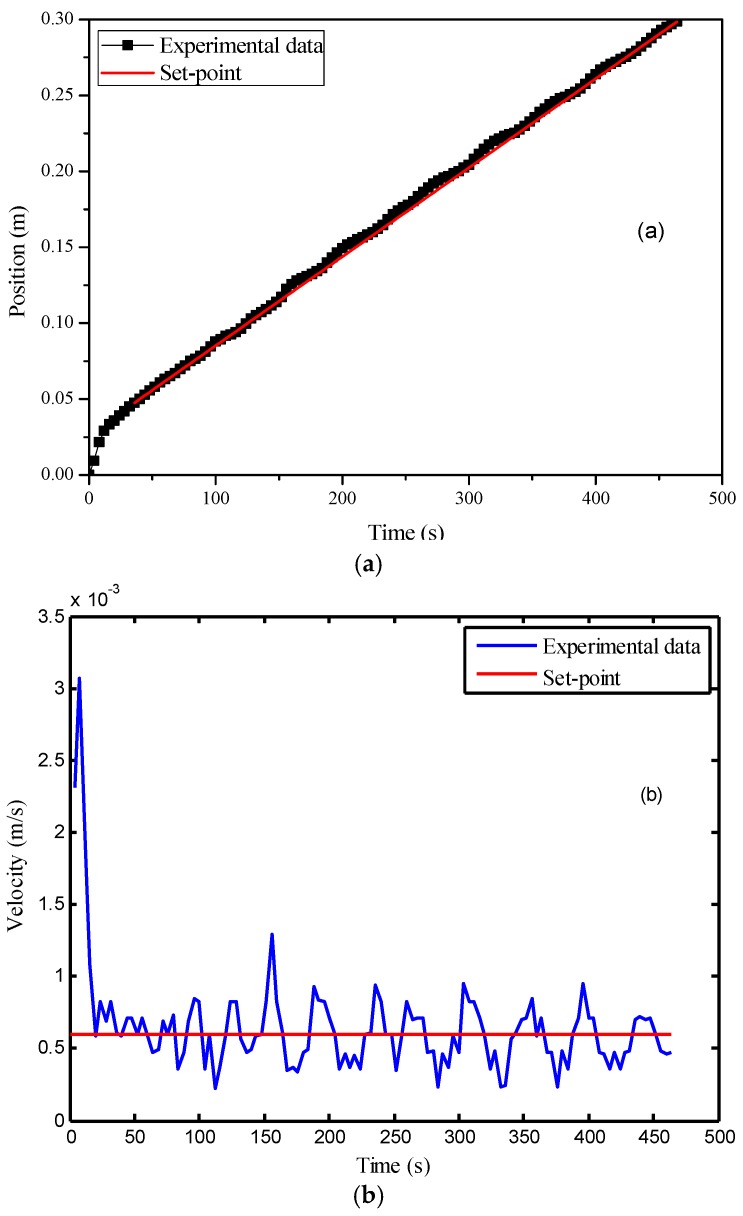
Control results of the PI (proportional–integral) controller: (**a**) time vs. flow front position; (**b**) time vs. flow front velocity.

Then, the PI parameters were obtained automatically using the auto-tuning function of Matlab/Simulink, where *K_c_* was selected as 55.9 and *τ_i_* was 0.125 s. After that, this controller was implemented for the velocity control of the flow front, where the set-point was selected as 6.0 × 10^−3^ m/s. In the experiment, the controller started up at the 36th second after the injection began. At this time point, the flow front velocity became smaller than the set-point for the first time. The control results are displayed in Figure 7. Figure 7a indicates that the average flow front velocity was very close to the set-point. However, from Figure 7b, it is observed that there existed significant oscillation in the variable’s profile. The standard deviation of the velocity after the 36th second was 1.9 × 10^−4^ m/s. Such control performance is unsatisfactory.

**Figure 8:**
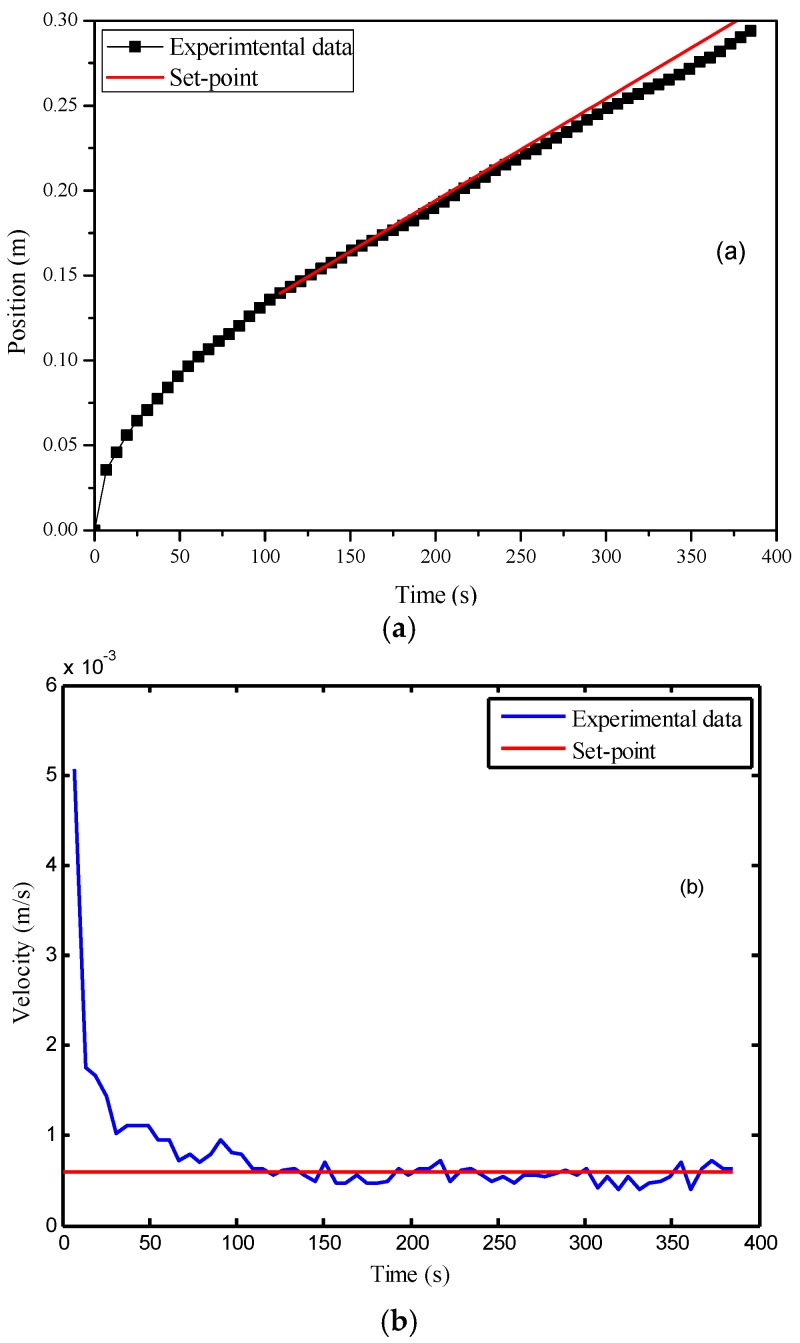
Control results of the MPC (model predictive control) with online permeability estimation: (**a**) time vs. flow front position; (**b**) time vs. flow front velocity.

For comparison, the MPC without the cascade structure was also implemented. In this experiment, the action time of the controller was later than that in the previous experiment. The possible reason is that the permeability values of the preforms used in these two experiments are different, causing a faster infusion at the beginning of the second experiment. The flow front velocity was larger than the set-point until the 162nd second. Therefore, the model predictive controller was activated at that time point. From Figure 8, it is obvious that the MPC led to a much smoother and less oscillatory profile of the controller variable. The standard deviation of the flow front velocity in the control was only 0.8 × 10^−4^ m/s, which is much smaller than that in the previous case, showing the benefit of the model predictive controller. It should be noted that there is a small offset between the control results and the set-point, which can be observed in Figure 8a. Such an offset was caused by a slight model mismatch.

**Figure 9:**
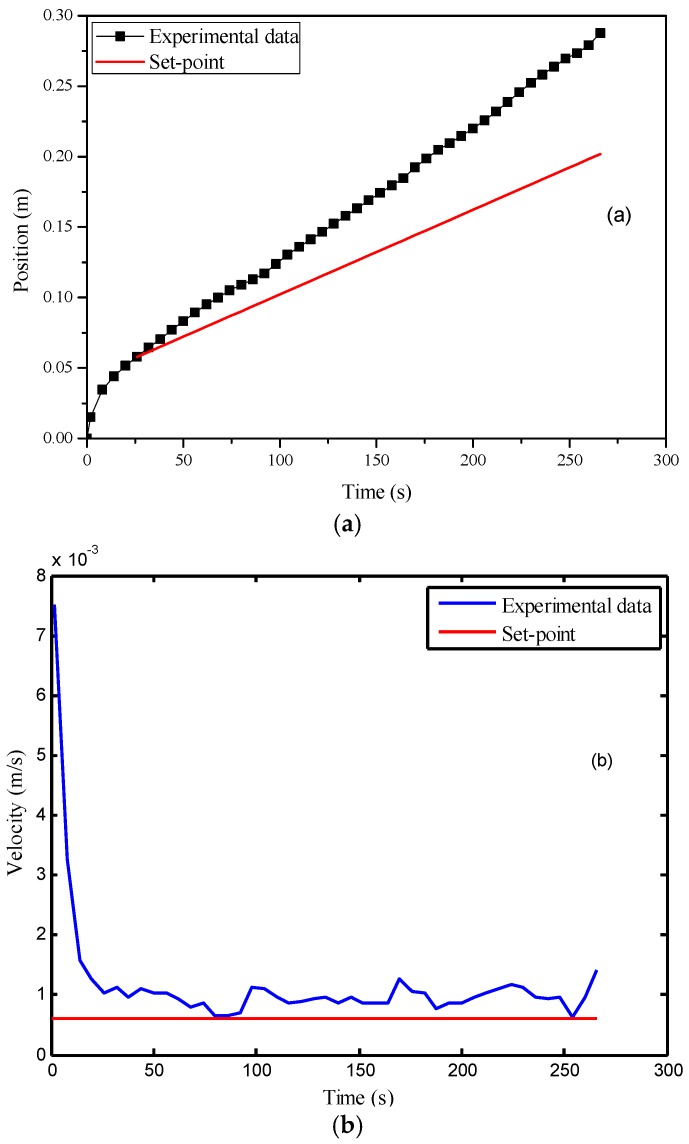
Control results of the MPC without online permeability estimation: (**a**) time vs. flow front position; (**b**) time vs. flow front velocity.

**Figure 10:**
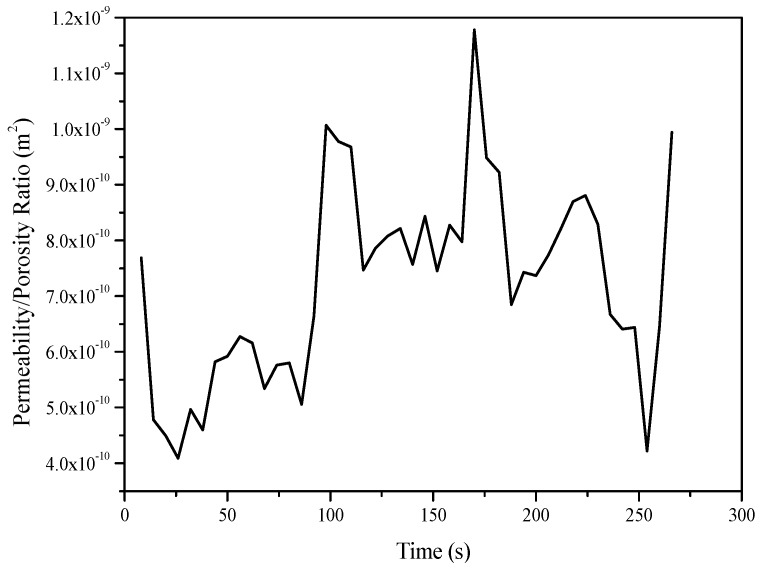
Local permeability/porosity ratio of the preform used in the experiment shown in Figure 9.

To better illustrate the advantage of integrating online estimation of the permeability/porosity ratio into the control strategy, two more experiments were conducted. In the first experiment, constant pressure control was implemented, while the method presented in [29] was adopted to estimate the equivalent permeability of the entire preform in an offline manner. The estimated permeability was 2.2 × 10^−10^ m^2^. The corresponding ratio between the permeability and the porosity was 4.0 × 10^−10^ m^2^, because the material porosity was known to be around 54.6%. In the second experiment, the fiber perform was made up in the same way, which consisted of nine layers of glass fiber mats. The permeability/porosity ratio estimated from the previous experiment was substituted into the meta-model for conducting the MPC to achieve a constant flow front velocity. However, the control results deviated from the set-point (6.0 × 10^−3^ m/s) significantly as shown in Figure 9. Figure 10 plots the values of the local permeability/porosity ratio of the preform used in this experiment which was estimated by the method introduced in [24]. Obviously, the real ratio largely deviated from the offline-estimated value, causing inaccurate prediction of the meta-model and poor control performance. By adopting the online estimation method proposed in this paper, such a problem can be avoided.

### 4.3. Performance of the Model-Assisted Cascade Controller

**Figure 11:**
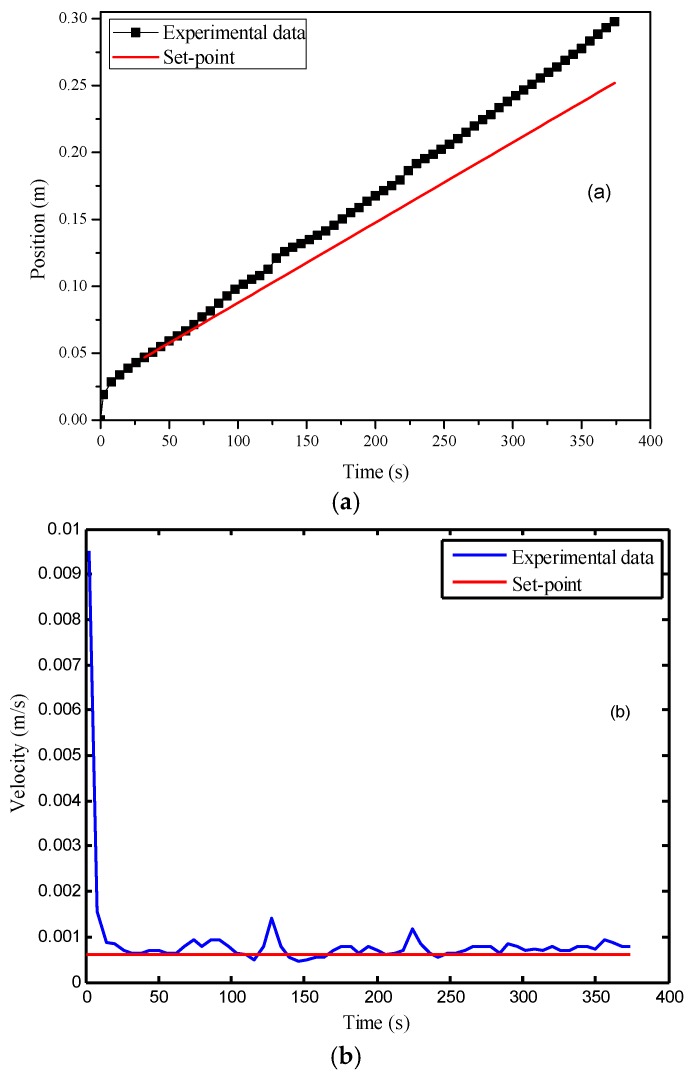
Control results of the MPC under model mismatch: (**a**) time vs. flow front position; (**b**) time vs. flow front velocity.

Although the MPC system with online permeability estimation can alleviate the model mismatch, it does not solve the problem completely as indicated by the control results in Figure 8. The following example demonstrates this issue more clearly. In this experiment, the meta-model was trained using the data generated using the process simulator by setting the ambient temperature to be 20 °C, where the resin viscosity was about 700 cps. Based on the meta-model, an MPC system was constructed to control the flow front velocity when the actual ambient temperature was about 30 °C and the resin viscosity was 457 cps. The change in viscosity led to large model mismatch, which further affected the control performance. As indicated in Figure 11, the over-estimation of the viscosity resulted in excessively high injection pressure and flow front velocity (7.2 × 10^−3^ m/s) larger than the set-point (6.0 × 10^−3^ m/s).

**Figure 12:**
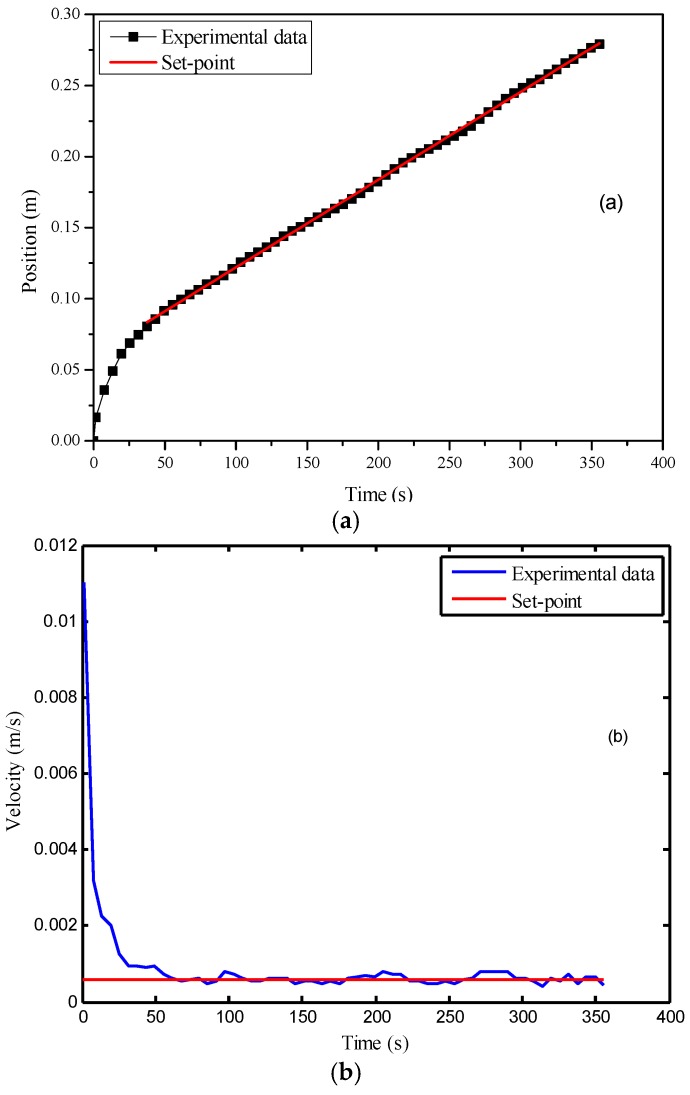
Control results of the model-assisted cascade controller under model mismatch: (**a**) time vs. flow front position; (**b**) time vs. flow front velocity.

Such problem can be solved by adopting the cascade control structure where a PI controller is added into the system to form an outer control loop. For parameter tuning of the PI controller, the entire MPC control system was regarded as a whole, and similar steps to those described in Section 4.2 were followed. Finally, *K_c_* was determined as 0.034, while *τ_i_* was selected as 0.90 s. Figure 12 shows that there is no significant deviation between the control results and the set-point despite the existence of model mismatch. Compared to that in Figure 11, the control performance was largely improved. The offset was eliminated by the integral action of the controller, while the standard deviation of the velocity after activating the controller was equal to 1.0 × 10^−4^ m/s.

## 5. Conclusions

In RTM processes, the ratio between permeability and porosity acts as a crucial parameter that affects both resin flow properties and final product quality. In this paper, based on the online estimation of the permeability/porosity ratio, a model-assisted control system is developed to stabilize the flow front in the mold in spite of the nonlinear and time-varying process characteristic. In the proposed control system, an RBF network meta-model is trained based on the data generated by a process simulator, which provides predictions of flow front positions. By optimizing the outputs of the meta-model, the injection pressure to be applied in the next step is determined. To further relieve the effects of the model mismatch, a cascade control structure can be adopted. The effectiveness of the proposed method is illustrated via experiments. To the best of our knowledge, it is the first time to introduce online estimation of permeability/porosity ratio and cascade structure into RTM flow control.

In the case studies, glass fiber reinforcement was utilized. However, the proposed method also works for the RTM processes using carbon fiber. In that case, the image contrast may be less significant for conducting binarization. Advanced image processing techniques, such as edge detection, can be adopted for flow front identification.

It is noted that, although the proposed method is implemented on a single-injection-gate RTM process, it can be extended to the control of a multi-gate system. The simplest strategy is to apply several model assisted controllers to manipulate the injection pressures supplied by different gates separately. Another issue to be discussed is that the estimation method developed in Section 3.2 only provides information about the fiber properties along the *x*-axis instead of the entire permeability tensor. As a result, when controlling a multi-gate system, model mismatch is unavoidable. However, such model mismatch can be compensated by the proposed cascade control strategy, and satisfactory flow control results can still be obtained.

## References

[1] Sozer E.M., Bickerton S., Advani S.G. (2000). On-line strategic control of liquid composite mould filling process. Compos. Part A Appl. Sci. Manuf..

[2] Bickerton S., Stadtfeld H.C., Steiner K.V., Advani S.G. (2001). Design and application of actively controlled injection schemes for resin-transfer molding. Compos. Sci. Technol..

[3] Hsiao K.-T., Advani S.G. (2004). Flow sensing and control strategies to address race-tracking disturbances in resin transfer molding. Part I: Design and algorithm development. Compos. Part A Appl. Sci. Manuf..

[4] Devillard M., Hsiao K.-T., Advani S.G. (2005). Flow sensing and control strategies to address race-tracking disturbances in resin transfer molding—Part II: Automation and validation. Compos. Part A Appl. Sci. Manuf..

[5] Nielsen D., Pitchumani R. (2001). Intelligent model-based control of preform permeation in liquid composite molding processes, with online optimization. Compos. Part A Appl. Sci. Manuf..

[6] Nielsen D.R., Pitchumani R. (2002). Closed-loop flow control in resin transfer molding using real-time numerical process simulations. Compos. Sci. Technol..

[7] Nielsen D.R., Pitchumani R. (2002). Control of flow in resin transfer molding with real-time preform permeability estimation. Polym. Compos..

[8] Lawrence J.M., Hsiao K.-T., Don R.C., Simacek P., Estrada G., Sozer E.M., Stadtfeld H.C., Advani S.G. (2002). An approach to couple mold design and on-line control to manufacture complex composite parts by resin transfer molding. Compos. Part A Appl. Sci. Manuf..

[9] Modi D., Correia N., Johnson M., Long A., Rudd C., Robitaille F. (2007). Active control of the vacuum infusion process. Compos. Part A Appl. Sci. Manuf..

[10] Lee D.H., Lee W.I., Kang M.K. (2006). Analysis and minimization of void formation during resin transfer molding process. Compos. Sci. Technol..

[11] Restrepo O., Hsiao K.-T., Rodriguez A., Minaie B. (2007). Development of adaptive injection flow rate and pressure control algorithms for resin transfer molding. Compos. Part A Appl. Sci. Manuf..

[12] Johnson R.J., Pitchumani R. (2006). Simulation of active flow control based on localized preform heating in a VARTM process. Compos. Part A Appl. Sci. Manuf..

[13] Johnson R.J., Pitchumani R. (2007). Flow control using localized induction heating in a VARTM process. Compos. Sci. Technol..

[14] Johnson R.J., Pitchumani R. (2008). Active control of reactive resin flow in a vacuum assisted resin transfer molding (VARTM) process. J. Compos. Mater..

[15] Matsuzaki R., Kobayashi S., Todoroki A., Mizutani Y. (2011). Control of resin flow/temperature using multifunctional interdigital electrode array film during a VaRTM process. Compos. Part A Appl. Sci. Manuf..

[16] Matsuzaki R., Kobayashi S., Todoroki A., Mizutani Y. (2013). Flow control by progressive forecasting using numerical simulation during vacuum-assisted resin transfer molding. Compos. Part A Appl. Sci. Manuf..

[17] Bender D., Schuster J., Heider D. (2006). Flow rate control during vacuum-assisted resin transfer molding (VARTM) processing. Compos. Sci. Technol..

[18] Li J., Fu X., Zhang C., Liang R., Wang B. (2009). Optimal injection design for resin transfer molding with in situ permeability measurement and process simulation. J. Compos. Mater..

[19] Ding L., Shih C., Liang Z., Zhang C., Wang B. (2003). In situ measurement and monitoring of whole-field permeability profile of fiber preform for liquid composite molding processes. Compos. Part A Appl. Sci. Manuf..

[20] Mogavero J., Sun J.Q., Advani S.G. (1997). A nonlinear control method for resin transfer molding. Polym. Compos..

[21] Berg J.M., Voller V.R. (1998). An identification and control strategy for a liquid composite molding process. Appl. Math. Model..

[22] Yenilmez B., Murat Sozer E. (2009). A grid of dielectric sensors to monitor mold filling and resin cure in resin transfer molding. Compos. Part A Appl. Sci. Manuf..

[23] Matsuzaki R., Kobayashi S., Todoroki A., Mizutani Y. (2011). Full-field monitoring of resin flow using an area-sensor array in a VaRTM process. Compos. Part A Appl. Sci. Manuf..

[24] Wei B.-J., Chang Y.-S., Yao Y., Fang J. (2016). Online estimation and monitoring of local permeability in resin transfer molding. Polym. Compos..

[25] Chou H.Z., Yang H., Hsu C.-C., Wei B.-J., Yao Y., Chang R.-Y. Through 3D numerical simulation and experimental visualization to study the resin transfer molding. Proceedings of the 29th Technical Conference of the American Society for Composites.

[26] Wang T.J., Wu C.-H., Lee L.J. (1994). In-plane permeability measurement and analysis in liquid composite molding. Polym. Compos..

[27] Ferland P., Guittard D., Trochu F. (1996). Concurrent methods for permeability measurement in resin transfer molding. Polym. Compos..

[28] Han K.K., Lee C.W., Rice B.P. (2000). Measurements of the permeability of fiber preforms and applications. Compos. Sci. Technol..

[29] Lee Y.J., Wu J.H., Hsu Y., Chung C.H. (2006). A prediction method on in-plane permeability of mat/roving fibers laminates in vacuum assisted resin transfer molding. Polym. Compos..

[30] Arbter R., Beraud J.M., Binetruy C., Bizet L., Bréard J., Comas-Cardona S., Demaria C., Endruweit A., Ermanni P., Gommer F. (2011). Experimental determination of the permeability of textiles: A benchmark exercise. Compos. Part A Appl. Sci. Manuf..

[31] Devillard M., Hsiao K.-T., Gokce A., Advani S.G. (2003). On-line characterization of bulk permeability and race-tracking during the filling stage in resin transfer molding process. J. Compos. Mater..

[32] Advani S.G., Sozer E.M. (2002). Process. Modeling in Composites Manufacturing.

[33] Kajero O.T., Thorpe R.B., Chen T., Wang B., Yao Y. (2016). Kriging meta-model assisted calibration of computational fluid dynamics models. AIChE J..

[34] Broomhead D.S., Lowe D. (1988). Multi-variable functional interpolation and adaptive networks. Complex Syst..

[35] Chen V.C.P., Tsui K.-L., Barton R.R., Meckesheimer M. (2006). A review on design, modeling and applications of computer experiments. IIE Trans..

[36] Lu J., Yao Y., Gao F. (2009). Model migration for development of a new process model. Ind. Eng. Chem. Res..

[37] Seborg D.E., Mellichamp D.A., Edgar T.F., Doyle F.J. (2010). Process. Dynamics and Control.

